# Tuberculous Spondylitis Following Kyphoplasty: A Case Report and Review of the Literature

**DOI:** 10.1097/MD.0000000000002940

**Published:** 2016-03-18

**Authors:** Chao-Yuan Ge, Li-Ming He, Yong-Hong Zheng, Tuan-Jiang Liu, Hua Guo, Bao-Rong He, Li-Xiong Qian, Yuan-Tin Zhao, Jun-Song Yang, Ding-Jun Hao

**Affiliations:** From the Department of Spine Surgery, Hong-Hui Hospital, Xi’an Jiaotong University College of Medicine, Beilin District, Xi’an, Shaanxi Province, China.

## Abstract

Tuberculous spondylitis of the augmented vertebral column following percutaneous vertebroplasty or kyphoplasty has rarely been described. We report an unusual case of tuberculous spondylitis diagnosed after percutaneous kyphoplasty (PKP).

A 61-year-old woman presented to our institution complaining of back pain following a fall 7 days before. Radiologic studies revealed an acute osteoporotic compression L1 fracture. The patient denied history of pulmonary tuberculosis (TB) and there were no signs of infection. The patient was discharged from hospital 4 days after undergoing L1 PKP with a dramatic improvement in her back pain. Two years later, the patient was readmitted with a 1 year history of recurrent back pain. Imaging examinations demonstrated long segmental bony destruction involving L1 vertebra with massive paravertebral abscess formation. The tentative diagnosis of tuberculous spondylitis was made, after a serum T-SPOT. The TB test was found to be positive. Anterior debridement, L1 corpectomy, decompression, and autologous rib graft interposition, and posterior T8-L4 instrumentation were performed. The histologic examination of the resected tissue results confirmed the diagnosis of spinal TB. Anti-TB medications were administered for 12 months and the patient recovered without sequelae.

Spinal TB and osteoporotic vertebral compression fractures are similar clinically and radiologically. Spinal surgeons should consider this disease entity to avoid misdiagnosis or complications. Early surgical intervention and anti-TB treatment should be instituted as soon as the diagnosis of spinal TB after vertebral augmentation is made.

## INTRODUCTION

Percutaneous vertebral augmentation with transpedicular injection of bone cement, including vertebroplasty and kyphoplasty, is effective and safe in the treatment of painful and osteoporotic vertebral compression fractures, since first described by Galibert et al in 1987.^1^ Complications of this procedure are not unusual and are mostly cement-leakage related, including spinal cord or nerve root compression, pulmonary, and cerebral embolism. Spinal infection of the augmented vertebral column has rarely been reported.^2–9^ However, tuberculous spondylitis following vertebral augmentation is even less. To the best of our knowledge, only 5 reports of tuberculous spondylitis following vertebral augmentation have been reported.^10–14^ Herein, we present an unusual case of tuberculous spondylitis diagnosed after percutaneous kyphoplasty (PKP). We highlight the clinical features and need for early diagnosis of this pathology. We also propose some probable reasons for its happening and some treatment principles.

## CASE REPORT

**FIGURE 1:**
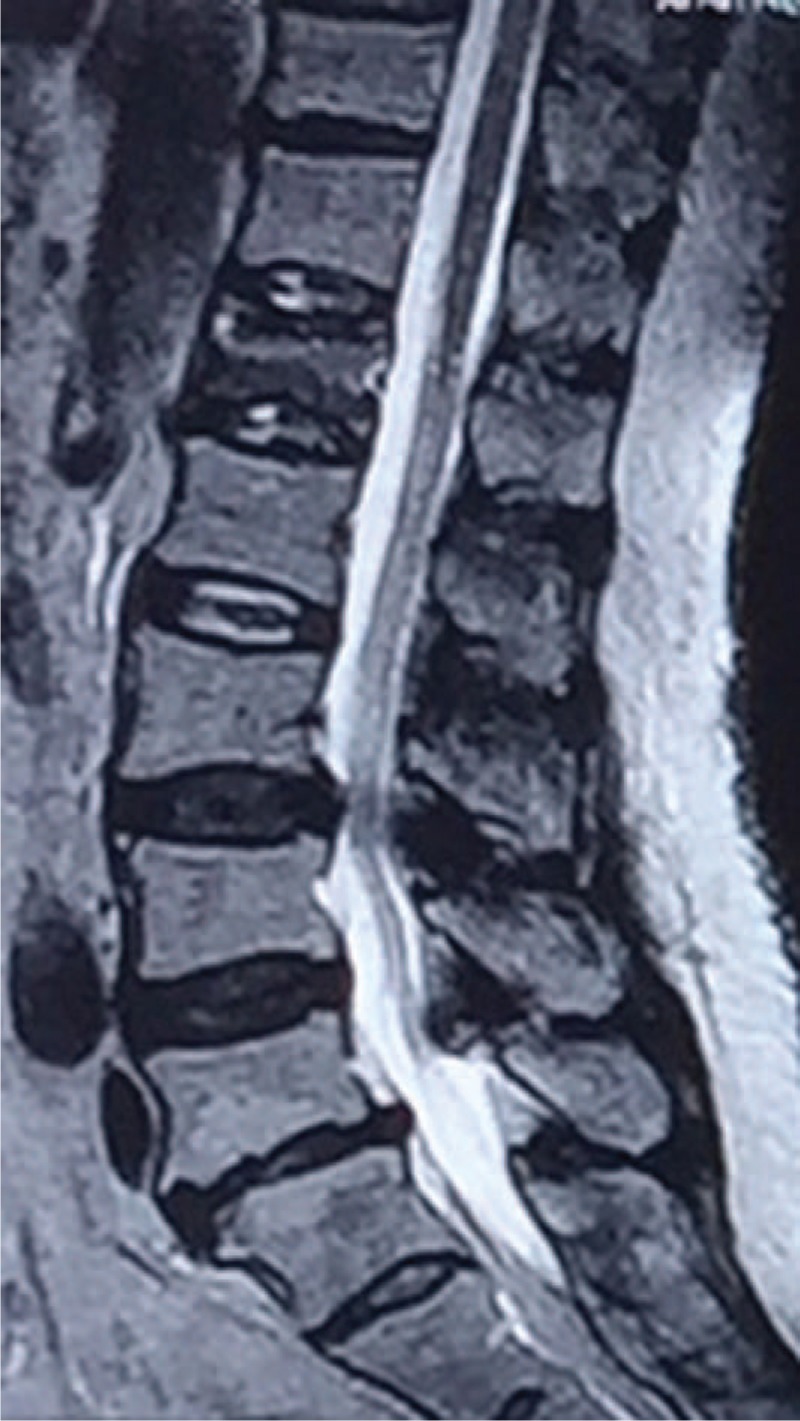
T2-weighted sagittal magnetic resonance imaging (MRI) of the lumbar spine showed an acute osteoporotic L1 compression fracture. MRI = magnetic resonance imaging.

**FIGURE 2:**
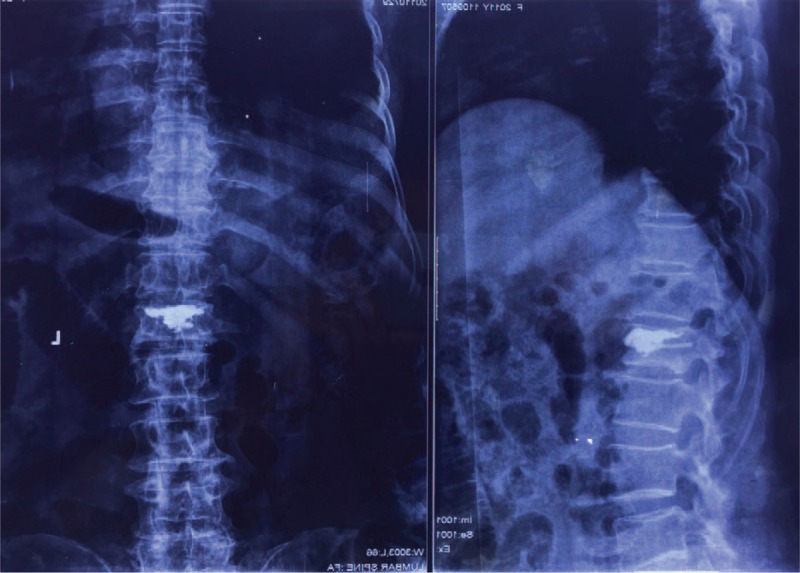
Postoperative anteroposterior and lateral radiographs after L1 percutaneous kyphoplasty (PKP). PKP = percutaneous kyphoplasty.

A 61-year-old woman with diabetes mellitus presented to our institution with a 1-year history of back pain. Her back pain worsened in the 2 months before admission and was accompanied by bilateral lower extremity weakness. She also complained of intermittent low-grade fever, night sweats, and weight loss. Two years ago, she presented to our institution complaining of back pain following a fall. Magnetic resonance imaging (MRI) revealed a fresh osteoporotic L1 compression fracture (Figure 1). At that time, there were no obvious abnormalities on examination and there was no evidence of an acute infection. The patient also denied a history of pulmonary tuberculosis (TB). She underwent L1 PKP under local anesthesia (Figure 2). Postoperatively, she was completely free of back pain and was discharged 4 days after surgery. The pathological examination of the specm obtained by biopsy needle during the surgery did not show any pathological cause of the fracture. One year postoperatively, she experienced recurrent back pain and underwent treatment at an outside hospital; the details of her treatment were obscure. Her back pain was somewhat relieved after therapy, but was aggravated in the 2 months before readmission, and was accompanied by bilateral lower extremity weakness.

On admission, she had a low-grade fever, restricted lumber motion with paravertebral muscle spasm and extensive thoracolumbar vertebral tenderness on examination. She had grade IV muscle strength in the bilateral lower extremities with shallow hypoesthesia. Her straight leg raising test was negative and the Bragard sign was seen. Her physiological reflexes, including light and deep reflection, were normal; there were no pathologic reflexes. The remainder of her physical examination was normal.

**FIGURE 3:**
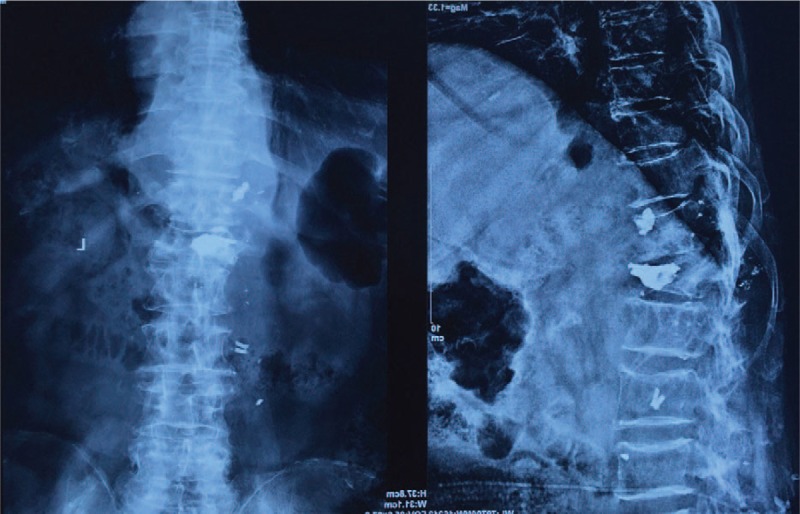
Two years later, radiograph of the same patient showed bony destruction of L1, and bone cement dissemination.

**FIGURE 4:**
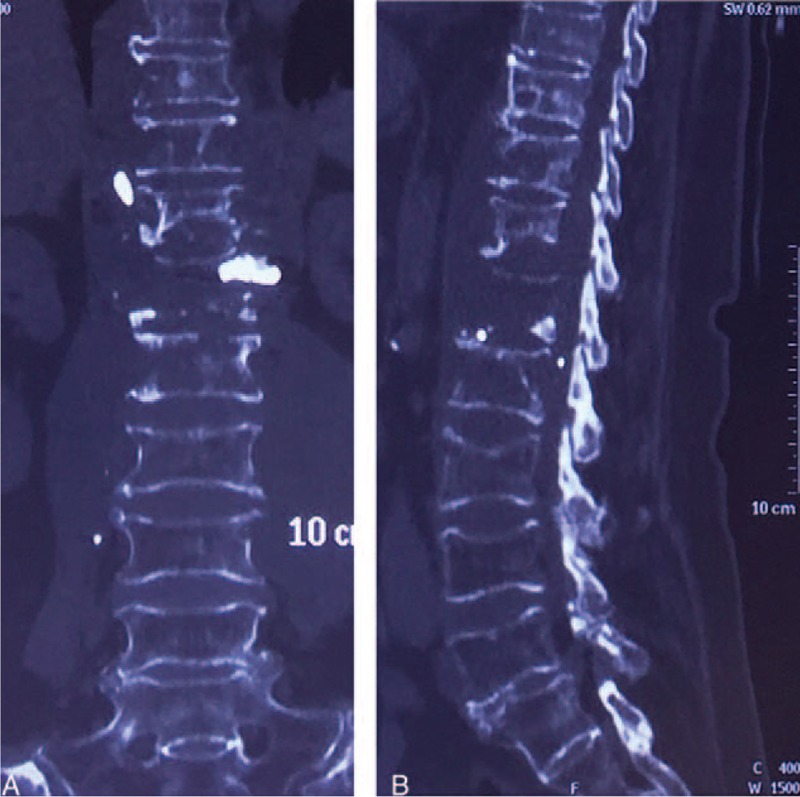
Thoracolumbar spine computed tomography (CT) images: coronal (A) and sagittal reconstructions (B). CT images of the thoracolumbar spine showed bony destruction of T9-L1 and paraspinal soft tissue widening. CT = computed tomography.

**FIGURE 5:**
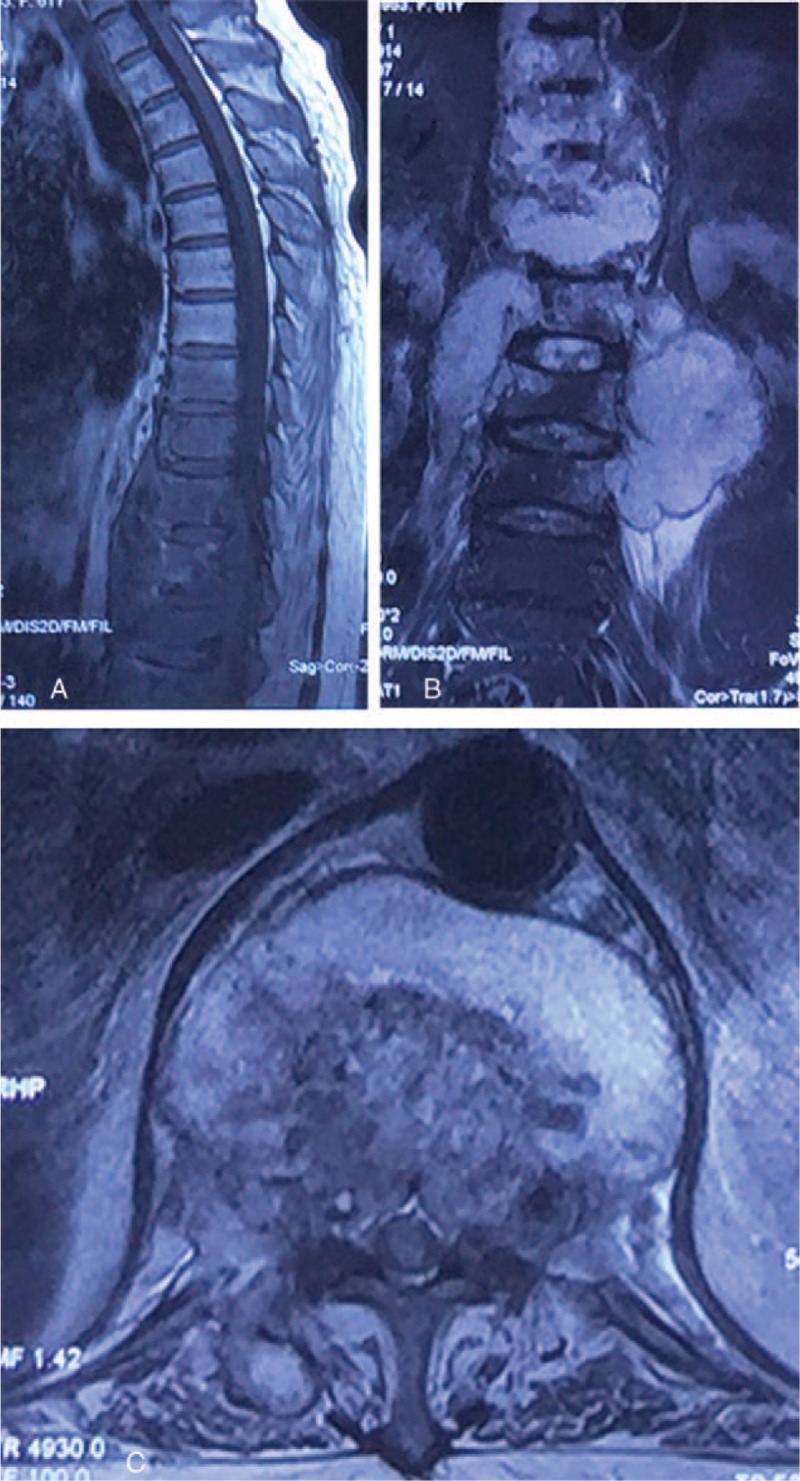
Thoracolumbar spine MRI images: sagittal reconstruction (A), coronal reconstruction (B), and axial reconstruction of L1 (C). MRI images of thoracolumbar spine indicated long segmental spondylitis involving T9-L3, with massive paravertebral abscess formation, with epidural abscess compressing corresponding levels of the dural sac. MRI = magnetic resonance imaging.

**FIGURE 6:**
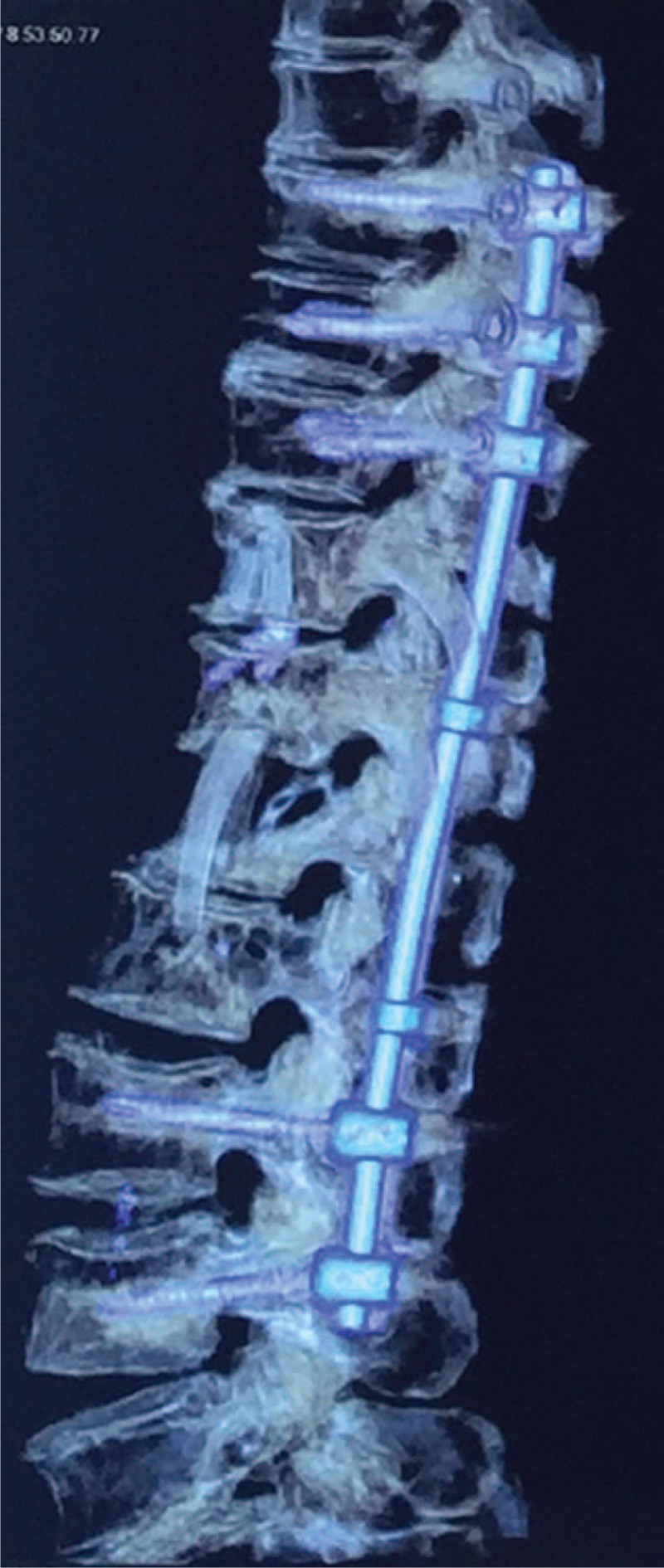
Postoperative 3D CT images after anterior debridement, L1 corpectomy, decompression, interposition of autologous rib graft, and posterior instrumentation of T8–L4. CT = computed tomography.

**FIGURE 7:**
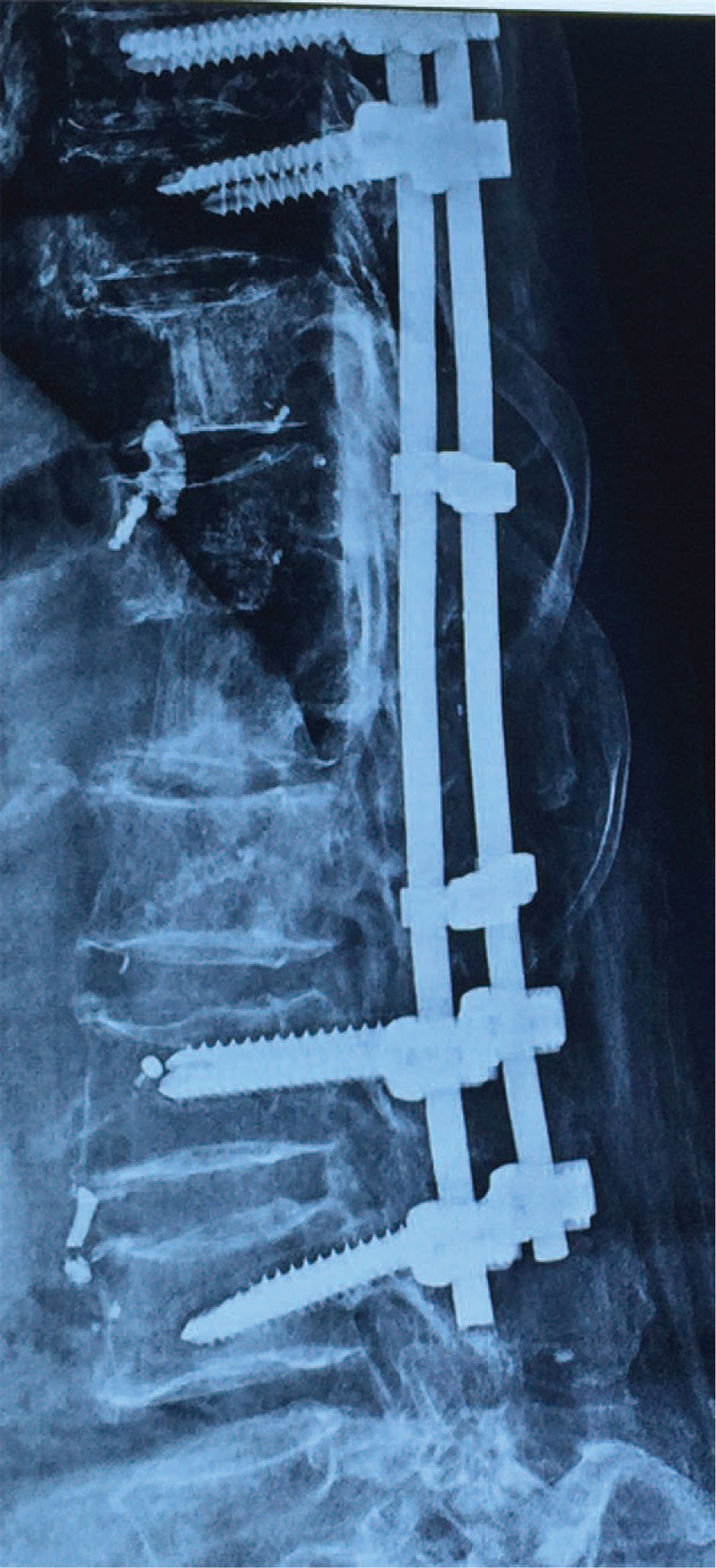
Twelve months after the surgery, lateral radiographs showed bony fusion of the rib graft and good position of the metallic implant.

Lumbar spine imaging demonstrated L1 bony destruction with bone cement dissemination (Figure 3). Computed tomography (CT) scan of the thoracolumbar spine showed T9-L1 bony destruction and paraspinal soft tissue widening (Figure 4). MRI showed long segmental T9-L3 spondylitis with massive paravertebral abscess formation. The epidural aspect of the abscess was compressing the corresponding levels of the dural sac (Figure 5). Although the patient denied a history of pulmonary TB, chest radiography and CT confirmed the diagnosis. Her white blood cell count was 7.06 × 10^9^/L with 73.61% neutrophils, and her erythrocyte sedimentation rate and C-reactive protein levels were elevated (82 mm/h and 40 mg/dL). A tentative diagnosis of tuberculous spondylitis was made after the serum T-SPOT. The TB test (specificity and sensitivity exceed 95%) was positive. Given the potential risk for increasing neurologic deficit and worsening clinical symptoms, anterior debridement, L1 corpectomy, decompression, autologous rib graft interposition, and posterior T8-L4 instrumentation were performed (Figure 6). Intraoperative bleeding exceeded 1800 mL; intraoperative blood transfusion in excess of 2000 mL was required. Histology of the infected tissue was consistent with tubercular spondylitis, including granulomatous inflammation with caseous necrosis and typical multinucleated giant cells. Adjuvant treatment with isoniazid (30 0 mg/d), rifampicin (600 mg/d), pyrazinamide (1500 mg/d), and ethambutol (675 mg/d) was given. Twenty days postoperatively, the patient was discharged with symptomatic improvement. She was continued on antituberculous medications for 12 months and recovered completely without sequelae. The lateral radiographs showed bony fusion of the rib graft and good position of the metallic implant at 12 months postoperatively (Figure 7).

The reporting of this case study was approved by the Ethics Committee of Hong-Hui Hospital, Xi’an Jiaotong University College of Medicine, Xi’an, China. In addition, written informed consent was obtained from the patient before data collection.

## DISCUSSION

PKP has gained widespread popularity and demonstrated clinical efficacy in the treatment of painful osteoporotic vertebral compression fractures.^15–22^ In spite of its rapid pain relief and safety, complications are not unusual and are most commonly associated with cement migration into the venous system, neural foramina, and posterior spinal canal, as well as cement emboli.^15,23,24^ Spinal infection after surgery has been reported, especially in immunosuppressive comorbidities such as diabetes and kidney transplantation.^2–4,6,7,9^ However, tuberculous spondylitis of the augmented vertebral column following percutaneous vertebroplasty (PVP) or PKP has rarely been described. To the best of our knowledge, only 5 reports of tuberculous spondylitis following vertebral augmentation exist (Table 1).^10–14^ Herein, we present a case of multifocal tuberculous spondylitis diagnosed after PKP.

**TABLE 1:**
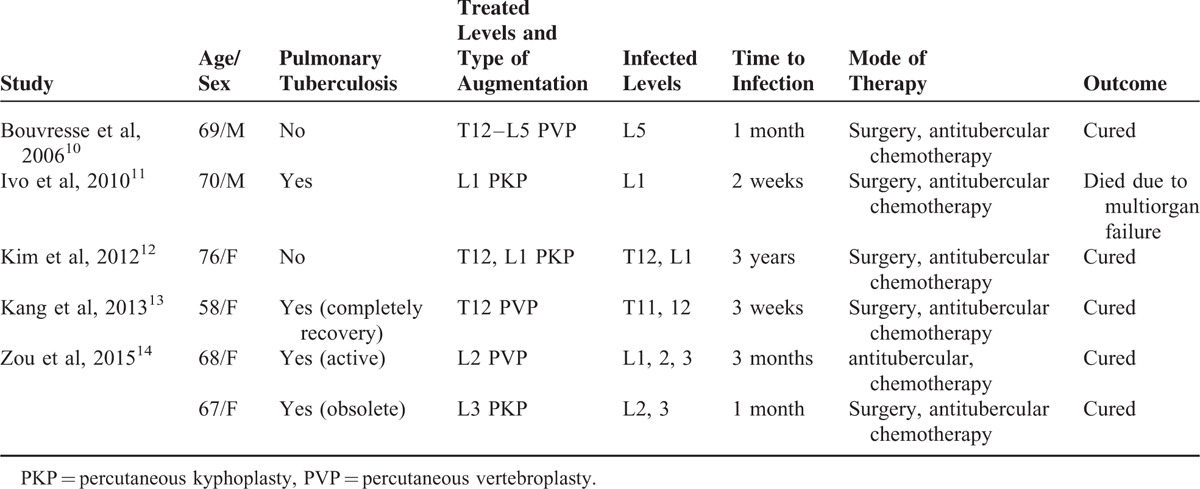
Summary of Previous Cases Reports of Tuberculous Spondylitis After Vertebral Augmentation

We found a common thread between the 5 previously reported cases: most of the patients were elderly (>67 years old, except for 1 who was aged 57 years). Our patient was also elderly (age 70 years). Additionally, most patients had immunosuppressive comorbidities. The patient reported by Bouvresse et al^10^ had a history of liver transplantation and had been treated with immunosuppressive agents including corticosteroids and tacrolimus. The patient reported by Ivo et al^11^ had an acute exacerbation of chronic obstructive pulmonary disease, type II diabetes mellitus, and esophagitis. Other patients also had comorbidities, such as hypertension or biliary cirrhosis.^12,14^ Our patient also had type II diabetes mellitus. Advanced age and comorbidities are both risk factors for spinal TB as its incidence is increasing in parallel with the growing numbers of immunocompromised patients.^11^

The exact underlying mechanisms through which TB can occur after PVP or PKP remain unclear.^14^ Thus far, the occurrence of tuberculous spondylitis after percutaneous vertebral augmentation can be explained in several ways. In the case of active pulmonary TB, hematogenous spread from the lungs to the vertebrae is the most probable mechanism,^11^ as reported by Ivo et al, Kang et al, and Zou et al.^11,13,14^ Meanwhile, in cases where patients did not have pulmonary TB at the time of injury, local reactivation (of an already present inactive tuberculous focus or migration of macrophages at the primary site of infection to the site of injury) initiated a new focus of tuberculous infection.^25^ The second explanation may account for the occurrence of spinal TB in our case because the patient had no history of pulmonary TB. In addition, spinal TB misdiagnosed as a vertebral compression fracture cannot be completely ruled out. Because spinal TB may clinically and radiologically resemble a compression fracture,^26,27^ the association of trauma and spinal TB may lead to a calamitous false diagnosis. In the case reported by Ivo et al,^11^ the patient was diagnosed with spinal TB and paravertebral abscess formation after PKP. Preoperative MRI revealed a subacute compression fracture; however, Chen et al^28^ preferred the diagnosis of the inflammation or infection of spine after carefully reviewing the preoperative MRI. In my opinion, the possibility of misdiagnosis is very high in this case.

Why is the surgical site, rather than other site, more easily infected by the tubercle bacillus? The term “locus minoris resistentiae” could offer an explanation. This term, first described by Agostoni,^29^ is defined as “a place of less resistance.” It can either designate an anatomic site more susceptible than another to physical injury during trauma, or the development of infection after an intentional traumatic event (surgery).^29–31^ This concept explains why tuberculous and nontuberculous mycobacterial infections arise at the site of injury or at the operative site. According to this concept, cement augmentation can be considered surgical trauma, which can lead to the initiation of a locus minoris resistentiae, with the subsequent development of tuberculous spondylitis.

The progression of spinal TB is very slow. The main symptom, back pain, is often too nonspecific to lead to a delayed diagnosis, resulting in a worse clinical prognosis. The exact diagnosis of the spinal TB usually requires the imaging examination and patients’ clinical presentation, together with a positive serology test. However, the diagnostic gold standard of the spinal TB, the pathologic findings, is usually too slow and often have a low sensitivity.^13^ Although MRI is currently the most accurate imaging study, clinical and radiologic features of tuberculous spondylitis and benign compression fracture are similar.^13,27^ However, tuberculous spondylitis and benign compression fracture may be difficult to differentiate in some cases, especially in the early stages. Therefore, the diagnosis of spinal TB is difficult and often delayed, resulting in a worse clinical prognosis.

Because of the rapid worsening of infection after kyphoplasty,^11,14^ it is very important to make a correct and early diagnosis of spinal TB following the augmented vertebral procedure. Serology testing for TB before invasive diagnostic procedures is recommended. The serology examination, also known as the T-SPOT test, has a reported sensitivity and specificity exceeding 95% and takes less time than microbiological and histologic examination of the specimen (which remains the gold standard). It has been widely used for fast and accurate diagnosis of TB in the outpatient setting. Percutaneous needle biopsy before vertebroplasty should be performed regularly in cases of osteoporotic vertebral compression fracture to rule out spinal malignancy or infection. In the case of risk factors for spinal TB, for example, immunosuppression, human immunodeficiency virus infection, or immigration from TB-endemic areas, histologic and microbiologic examination including microscopy, culture, and PCR analysis is of utmost importance to avoid delays in establishing the correct diagnosis and specific treatment.^11,32^ In addition, detailed history-taking focusing on a history of TB provides invaluable information that can hasten the diagnosis.

Antitubercular medication should be initiated immediately, once the diagnosis of spinal TB is established. In previous reports, first-line treatment of TB following PVP or PKP is surgical debridement with or without instrumented stabilization.^10–14^ However, this infection may respond favorably to conservative treatment, especially in patients without extensive bony destruction, massive paravertebral abscess formation, and neurologic deficits.^33^ Zou et al^14^ reported a 68-year-old patient, not a candidate for surgery because of acute pulmonary infection, who was given adjuvant chemotherapy comprised of isoniazid, rifampicin, pyrazinamide, and ethambutol. Her clinical symptoms were alleviated and she was eventually discharged from the hospital without surgical intervention. However, in the case of neurologic impairment or progressive infection unresponsive to antibiotic treatment, if medical treatment fails with increasing inflammatory markers and rapid worsening of the patient's clinical condition, anti-TB medication and extensive surgical debridement (including resection of the TB-infected focus and 360° spinal reconstruction) is in accordance with current treatment standards.^5,9,34^

The insertion of a metallic implant in spinal TB is to provide mechanical stability. Some experimental studies have suggested that spinal TB, unlike bacteria, have low adherence to stainless steel and form less of a polysaccharide biofilm.^35,36^ Therefore, the use of implants in the presence of spinal tuberculous spondylitis is theoretically safe. Indications for instrumented stabilization in spinal TB can be categorized as the following: 3 columns of vertebrae are infected, long-segment disease with a bone graft >5 cm after debridement, and when surgical correction of a kyphosis is performed.^35^ In our case, the spinal TB was a long-segment focus involving T9-L1, with massive paravertebral abscess formation. Combined surgery consisting of anterior debridement, L1 resection, decompression, and interposition of autologous rib graft and posterior instrumentation of T8-L4 was performed, and the patient recovered.

## CONCLUSION

This case illustrates that although rare, activated tuberculous spondylitis by *Mycobacterium tuberculosis* should be considered a possible complication of PVP or PKP, especially if the patient has a history of previous pulmonary TB. Careful preoperative diagnostic trials including the serology T-SPOT test, needle biopsy, and PCR analysis for *Mycobacterium tuberculosis* should be performed regularly. As the diagnosis of tuberculous spondylitis is established, if conservative anti-TB medication fails, resection of the infected bone–cement complex and instrumented stabilization is indicated.
